# Integration in the Vocational World: How Does It Affect Quality of Life and Subjective Well-Being of Young Adults with ASD

**DOI:** 10.3390/ijerph120910820

**Published:** 2015-09-02

**Authors:** Eynat Gal, Efrat Selanikyo, Asnat Bar-Haim Erez, Noomi Katz

**Affiliations:** 1 Occupational Therapy Department, University of Haifa, Haifa 31905, Israel; 2 Ono Academic College, Occupational Therapy Department, Research Institute for Health and Medical Professions, Kiryat Ono 55000, Israel; E-Mails: efratselanikyo@gmail.com (E.S.); aaerez@gmail.com (A.B-H.E.); noomi.katz@ono.ac.il (N.K.)

**Keywords:** quality of life, well-being, work, autism spectrum disorders (ASD)

## Abstract

This study aimed to assess whether the perception of quality of life (QOL) and subjective well-being (SWB) of young adults with autism spectrum disorders (ASD) is affected by participation in a comprehensive program. Participants included 25 young adults with ASD who participated in the “Roim Rachok Program” (RRP), where they were trained to become aerial photography interpreters. Following the training period, they served in a designated army unit where they practiced their newly acquired profession. The participants filled out two questionnaires, (a) Quality of Life (QOL-Q) and (b) Personal Well-being Index (PWI), at three points of the intervention: (a) before the course, (b) at the end of the course, and (c) six months after integrating in the designated army unit. Wilcoxon signed ranks tests were used to assess the differences between the reported QOL and SWB at the three points of time. The results suggest that there were no significant differences at the end of the course, compared to its beginning. However, there were significantly improved perception of QOL and SWB during the period between the end of the course and six months after starting work. The results of this study highlight the importance of tailored vocational programs that are adapted to the unique needs and strengths of individuals with ASD.

## 1. Introduction

A primary aspiration of adolescents and young adults upon completing their studies is to successfully integrate into the world of work [1]. Work has been defined as productive activities performed with or without payment, and includes preparatory activities, as well as the generation and provision of services and activities that contribute to the development and advancement of the society and the individual [2]. One’s ability to work, according to the *International Classification of Functioning, Disability and Health* [3] is considered to be significant for one’s health, quality of life, and well-being.

The concept of quality of life (QOL) includes (a) a conceptual framework for assessing personal outcomes; (b) a social construct that guides program practices and quality improvement (QI) strategies; and (c) a criterion for assessing the effectiveness of those practices and strategies [4]. It includes objective and subjective indicators, a broad range of life domains, and individual values, affected in a complex way by the person’s physical health, psychological state, level of independence, social relationships, personal beliefs and their relationship to salient features of their environment [5].

Developmental disabilities affect all of the above-mentioned constructs and therefore pose a great challenge to one’s quality of life.

Few disorders pose a greater threat to the quality of life and psychosocial well-being of individuals and their caregivers than autism spectrum disorders (ASD) [6], a lifelong disorder characterized by restricted and repetitive patterns of behavior and social communication deficits [7]. According to the Diagnostic and Statistical Manual of Mental Disorders, 5th Edition (DSM-5), ASD has three levels of severity, determined by the level of impact of the communication and behavioral symptoms and the amount of support required. People who are considered to be at “level 1”, which is the least severe, are characterized by a significant deficit in social skills, expressed by inappropriate responses in social situations, difficulty initiating social interaction, limited interest in forming social relationships and by ritualistic and repetitive behaviors that cause significant difficulties in one or more contexts, but also with a high intellectual level and the least requirements for support [7].

Although integration into the free labor market may represent a challenge for anyone, people with developmental deficits in general and with ASD in particular, encounter a variety of significant difficulties when approaching the stage at which they have to find and maintain a job.

Indeed, most people with ASD, including those who are cognitively able, have difficulty participating in everyday life occupations across the life span, with work presenting a unique challenge. Cognitively able young adults with ASD, despite having average and above average intelligence, and the capability of working in competitive employment, often face substantial difficulty finding and retaining paid employment [8], and many are not employed or are employed in jobs that do not meet their abilities and preferences [9]. Such difficulties may affect their perception of well-being and quality of life. These days, the concept of quality of life is used to plan and measure the success of intervention and rehabilitation programs. 

ASD is typically diagnosed in early childhood, has a lifelong course and currently has no cure, and the available interventions require a tremendous investment of time, money and energy on the part of the child’s caregivers. It is not surprising, therefore, that past research has suggested that mothers of young children with ASD exhibit higher levels of stress, mental health symptoms, and marital discord than mothers of children with other developmental disabilities [10,11,12]. However, in contrast to the abundance of studies focused on the impact of caring for children and adolescents with ASD, in general, and specifically on the well-being of parents of children with ASD [13,14,15], very little research has focused on the well-being of those with ASD themselves. 

Since there is currently a lack of knowledge regarding the subjective perception of quality of life of people with developmental disabilities and specifically with people on the autism spectrum, we studied whether an adapted and safe workplace for young adults with ASD results in an enhanced perception of well-being. 

In Israel, military service is compulsory at the age of 18 (parallel to entering college in the United States), and represents both a major milestone in the lives of young adults, and the first step in the vocational world. However, people with ASD, including many of those who are cognitively able, are automatically discharged of military service. This means that after high school, they are often left isolated from their previous social life and occupations, and feel helpless in facing their future. The “Roim Rachock” Program (RRP) (“Viewing Far” in Hebrew) is an innovative program, designed to integrate cognitively able young adults with ASD first into the army work force, and later into the free market. The program is based on the vision that certain professions could be identified that would match the special characteristics of people with ASD. To date, four such professions have been identified: (a) aerial photography interpretation, (b) software quality assurance (QA), (c) information processing, and (d) optics technicians (repairing optical equipment as binoculars or foresights). The RRP includes two main stages: (1) a three-month course operated by an interdisciplinary team, which includes army officers and instructors, an occupational therapist, a speech therapist and an art therapist; and (2) inclusion in designated army units and serving as aerial interpreters along with other soldiers who do not have developmental disabilities (see more in the intervention program). 

The aim of the current study was therefore to assess whether RRP, a vocational program tailored to the skills and strengths of individuals with ASD, affects their quality of life and subjective well-being. We specifically aimed to assess which part of the program contributed most to their satisfaction from their QOL. 

## 2. Method

### 2.1. Study Design

This study followed a pre-test/post-test design, with the purpose of investigating perception of QOL and subjective well-being among young adults with ASD, who took part in a professional training and employment placement (the RRP program). 

### 2.2. Study Groups

The twenty-five individuals with ASD, who participated in the RRP and took part in two identical aerial photography interpretation courses, participated in this study. Participants included 24 men (96%) and one woman, ages 18 to 22 (mean = 19.08). All participants had a Pervasive Developmental Disorder (PDD) diagnosis from a licensed professional according to DSM IV, including 9 )36%) diagnosed with Pervasive Developmental Disorder- Not Otherwise Specified (PDD-NOS); 2 (8%) diagnosed with ASD and 14 (56%) diagnosed with Asperger’s Syndrome. ASD diagnosis was confirmed with Social communication questionnaire (SCQ). 

Ten (40%) participants previously studied in regular high school settings; 1 (4%) studied in a regular school setting with an assistant; 10 (40%) studied in a special education class within regular school settings; 4 (16%) studied in a special school setting; and 1 (4%) dropped out of school at the age of 16. In accordance with the Israeli special education law (the right for education up to age 21), years of education varied from 10 to 15 (Mean = 12.27). All participants but one (the participant who dropped out of school) completed matriculation tests, in full or part, which, in Israel are considered to be one of the criterion for acceptance to university. They also went through extensive screening testing to define their language, writing and visual processing abilities, and were accepted to the program, only upon passing these tests. In addition, they were all approved by the army mental health officials for voluntary army service. 

### 2.3. Instruments

#### 2.3.1. Quality of Life Questionnaire (QOL-Q)

The QOL-Q [16] is a 40-item rating scale originally developed for persons with intellectual disabilities, but used with ASD as well, and designed to measure overall QOL. The scale is administered in interview format and yields data regarding overall QOL, consisting of scores from four sub-scales. The four subscales include: satisfaction (e.g., do you have more or fewer problems than other people?), competency/productivity (e.g., do you feel your work is worthwhile and relevant?), empowerment/independence (e.g., who decides how you spend your money?) and social belonging/community integration (e.g*.*, how satisfied are you with the clubs and organizations to which you belong?). Each subscale contains 10 items, scored on a three-point Likert scale. Scores on the total QOL-Q range from 40 to 120, and a higher score represents a higher level of overall quality of life. The QOL-Q has good psychometric properties with a test–retest coefficient of 0.87 and with a Cronbach’s alpha coefficient of 0.90 for the total scale [16].

#### 2.3.2. Personal Wellbeing Index (PWI)

The PWI scale contains eight items, each one corresponding to a quality of life domain: standard of living, health, life achievement, relationships, safety, community-connectedness, future security, and spirituality/religion [17,18]. The index uses a 0–10 Likert response scale, with response options ranging from “completely dissatisfied” to “completely satisfied”. Each of the eight domains (items) can be analyzed as a separate variable, or the eight domain scores can be totaled to yield an average score, which represents “subjective well-being”. It is designated for use with the general adult population, aged at least 18 years. These eight domains are theoretically embedded, as representing the first level deconstruction of the global question: “how satisfied are you with your life as a whole?” The scale shows good internal consistency with Cronbach’s alpha between 0.70 and 0.85 in Australia and other countries. Published normative data regarding PWI exists in Australia, Asia, and few western cultures, however, not in Israel, so they are therefore related to as a point of reference only.

### 2.4. Procedure

After obtaining approval from the Research Ethics Board of the Ono Academic College, the participants received an explanation about the study and were asked to sign consent forms. They were notified that their participation in the study was voluntary and could be terminated at any stage of the study without consequences. Next, letters of explanation were sent to the parents and a parental signed consent form was obtained.

**Figure 1:**
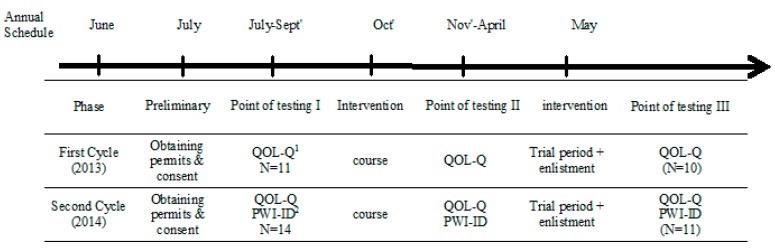
Research time line and procedure. Notes: **^1^** Quality of Life Questionnaire; ^**2**^ Personal Wellbeing Index.

The testing was carried out at three points: (1) prior to the aerial photography interpretation course; (2) following the course; and (3) after six months of being integrated in the army unit (see Figure 1 for research design). At each point of data collection, participants were asked to complete the QOL-Q and the PWI questionnaires. Data collection was conducted by the last author and a registered occupational therapist.

The number of participants’ varied for two reasons: (1) along the training period several participants did not meet the requirement for army service and were placed in a similar work environment in the civil sector. (2) For the PWI, unfortunately, data was not collected in the first and second testing points of the first course as the questionnaire was not yet included in data collection (see Figure 1). 

### 2.5. Intervention Program

The study included two program phases: (a) The aerial photography interpretation course, and (b) a 6 month army phase. The actual program includes a much longer army phase, but the current study addressed the first 6 months. 

#### 2.5.1. Aerial Photography Interpretation Course

The aerial photography interpretation courses were designed to simulate the working environment in the army as much as possible. Each course was carried out for three months, five days a week, from 9:00 a.m. to 3:00 p.m. The courses were operated by an interdisciplinary team that included both army personnel and civil health and psychological professionals. Course contents were composed of two main learning domains: (1) decoding aerial photography (e.g., identifying civil infrastructure); and (2) integration within the army working environment. The latter domain focused on teaching working skills (e.g., working independently according to a checklist), Instrumental Activities of Daily Living (IADL) skills (e.g., using public transportation), communication skills (e.g., how to ask for help), social skills (e.g., how to get to know a new co-worker), self-advocacy skills (e.g., one’s rights and obligations as a soldier) and how to establish hierarchal-based communication with army commanders. 

#### 2.5.2. Army Phase

The army phase of the “Roim Rachok Program” (RRP) includes two parts: a trial, followed by signing up for one year of service with the yearly option of extension, for up to three years.

The trial lasted for three months, and its purpose was to allow the participants to experience the future working environment in the army before committing to actual army service. During the trial period, participants are integrated into the army as civilian volunteers, but serve in their designated job, in the “real” work environment, namely the designated army unit. The army phase in the current study included six months of actual work in the designated unit; three of them of “trial time” followed by three months of formal army service. 

### 2.6. Data Analysis

**Figure 2:**
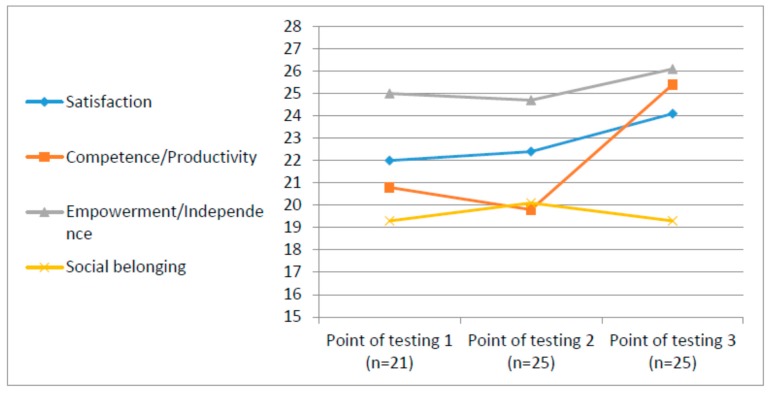
Comparison of personal perception of quality of life by QOL-Q at three different points of testing Point of Testing 1—at the beginning of the course; Point of Testing 2—at the end of the course; and Point of Testing 3—after six month of integration in army unit.

**Figure 3:**
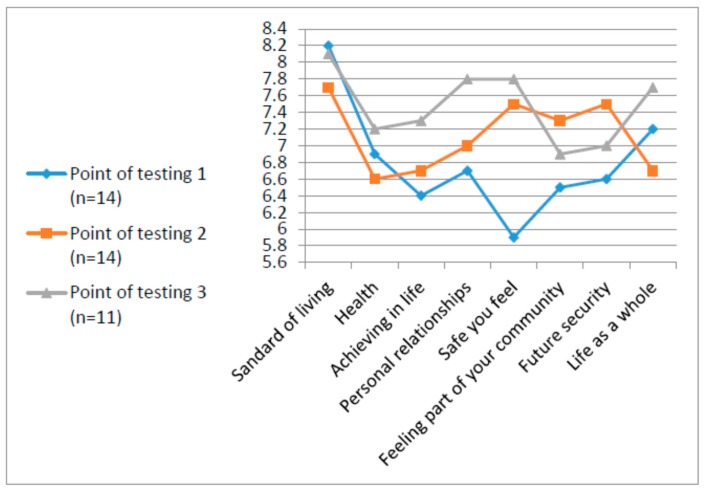
Comparison of personal perception of well-being by PWI in three different points of testing. Point of testing 1—at the beginning of the course; Point of testing 2—at the end of the course; and Point of testing 3—after six month of integration into army unit.

Statistical measures (means and standard deviation) of the PWI and the QOL-Q were analyzed in order to describe each variable distribution, as shown in Table 1, Table 2, Figure 2 and Figure 3. Wilcoxon signed ranks tests were used to assess the differences for both measures between the three time points. Friedman Test was used for the PWI analyses, which is based on a small N. 

## 3. Results

### 3.1. Quality of Life Questionnaire (QOL-Q)

Most of the means in each domain of the questionnaire range from 17 to 26 out of a possible 30 points, with SDs ranging from 1.39 to 5.73, demonstrating large variability (see Table 1).

Results of the QOL-Q suggest that there were no significant differences in participants’ perception of their subjective quality of life at the end of the course compared to its beginning (*n* = 25) in all areas assessed by the questionnaire, namely satisfaction, competence/productivity, empowerment/independence, and social belonging. However, significant statistical differences were found from the end of the course (T2) to six months post-integration into the unit (T3) (*n* = 21). Total score (*z* = −2.86, *p* < 0.002); Satisfaction (*z* = −2.09, *p* < 0.019); Health (*z* = −1.81, *p* < 0.035); and Productivity (*z* = −3.61, *p* < 0.000), can also be seen in Figure 2. In all areas, the participants indicated higher perceived QOL except for the sense of social belonging. In this domain, an increase was noted immediately after the course (*z* = −2.17, *p* < 0.015), but after six months in the army, it returned to its previous level. 

**Table 1:** Means and standard deviations and Wilcoxon analysis of QOL-Q at three points.

QOL Domains	M (SD)	M (SD)	M (SD)	*Z*	*p*
Satisfaction	22.00 (3.14)	22.36 (3.50)	24.10 (3.21)	−2.09	0.019
Competence/Productivity	20.76 (4.65)	19.84 (4.84)	25.38 (2.78)	−3.61	0.000
Empowerment/Independence	24.96 (2.89)	24.68 (2.97)	26.14 (2.83)	−1.81	0.035
Social belonging	19.28 (4.24)	20.08 (4.86)	19.29 (3.73)	−2.17	0.015

T1 = prior to the course; T2 = at end of the course; T3 = after six months of work. SD = Standard deviation.

### 3.2. Personal Well Being Index (PWI)

Data on the PWI is available for the second course only, at the three points of testing (T1 and T2, *n* = 14; T3, *n* = 11). Means of performance in the seven domains and the general WBI item range from 5.93 to 8.21 (SDs ranges from 2.06 to 2.71), as shown in Table 2. 

The main domain that showed significant improvement between points of testing, T1 to T2 (pre and post course, n=14) was the satisfaction from personal safety (*z* = −2.42, *p* < 0.008). As can be seen in Figure 3, although most domains did not reach statistical significance, it is interesting to note the change trend in some domains. For example, the domains of future security and connectedness to the community, increased immediately after the course. In contrast, satisfaction from personal relationship and self-determination increased at the later stage, namely the employment stage. 

**Table 2:** Means and standard deviations of PWI at three points of testing (T1, T2, T3).

Time of Testing	T1 *n* = 14	T2 *n* = 14	T3 *n* = 11
**QOL Life Domains**	**M (SD)**	**M (SD)**	**M (SD)**
Life as a whole	7.21 (2.08)	6.86(2.03)	7.73 (1.42)
Standard of living	8.21 (1.67)	7.71 (1.14)	8.09 (1.51)
Health	6.86 (2.60)	6.57 (2.62)	7.18 (2.09)
Life achievement	6.43 (1.74)	6.71 (2.33)	7.27 (1.27)
Relationships	6.71 (1.68)	7.00 (2.22)	7.82 (0.87)
Safety *	5.93 (2.20)	7.50 (2.35)	7.82 (1.89)
Community-connectedness	6.50 (1.87)	7.29 (1.98)	6.91 (1.97)
Future security	6.57 (2.34)	6.86 (2.63)	7.00 (1.34)

Notes: Only significant difference between T1 and T2 for the safety variable. * *z* = −2.42, *p* < 0.008 (*n* = 14). T1 = prior to the course; T2 = at end of course; T3 = after six months of work.

## 4. Discussion

Individuals with ASD who are cognitively able, can work successfully in community businesses [19], but the majority of them have substantial difficulties in finding and in maintaining paid employment [20], and many of them are not employed, or are employed in jobs that do not meet their abilities and preferences [9]. This discrepancy may often result in frustration and a sense of worthlessness, therefore affecting the self-perception of quality of life and well-being of many young adults with ASD who face the stage of needing to integrate in the vocational world. 

The RRP is a unique vocational program aimed at integrating young adults with ASD into the army in meaningful jobs that match their abilities. It is a comprehensive vocational program that includes training, placement and support in the workplace. Participants learn a profession, in the case of the current study, aerial interpretation, which utilizes the good visual perception and analytic skills that characterize many people with ASD [21], while concurrent support is provided to address the difficulties in various social and functional skills that are considered to be challenging to those with ASD [7]. The current study aimed, therefore, to assess whether participation at the RRP affected the participants’ perception of their quality of life and subjective well-being. Specifically, we aimed to assess changes in satisfaction from various life domains, and whether there was a positive change due to (a) the course phase and (b) the integration in the army unit phase. 

### 4.1. Quality of Life Along the Vocational Program

The QOL-Q is a measure that was developed, initially, for selected population (learning disabilities), but is also recommended for use in other populations such as ASD [22]. It targets specific four QOL domains: (a) satisfaction, (b) competence/productivity, (c) empowerment/independence, and (d) social belonging.

Interestingly, the results concerning QOL indicated significant statistical differences only at the third measuring point, namely, following integration into the designated army unit. The participants, young adults with ASD, reported satisfaction with general life, with their competency in their occupation and their perceived independence and empowerment. This trend was not apparent in the second point of measure, following the training phase (the course). 

During the first phase, in the course, participants are accepted into a prestigious program, meaning that their skills are acknowledged via objective tests and by subjective interviews. They learn the specific tasks of the profession within a segregated environment, where they have the chance to create social relationships with people who have similar profiles. Their Activities of Daily Living (ADL) and IADL skills improve and their independence is therefore enhanced. They acquire a profession that is adapted to their skills and that is highly regarded in the community, and they learn self-advocacy skills. Nevertheless, none of these aspects seemed to affect the QOL of the participants. However, following six months in the newly acquired profession within the designated army unit, the participants reported significantly better QOL in all questionnaires’ domains.

Possibly, the course itself reminded the participants of other vocational programs that they attended at earlier stages or was very challenging. In addition, at this stage, participants learned within a segregated environment, and were not yet integrated with typically developed young adults and within the actual target occupation, as they were at the army stage. 

In the second phase of the program, however, the participants were included in the designated army unit. This means that they were formally drafted into the army and started their service. At this stage, they were included in a unit, which was designed to meet their needs, with three army commanders assigned for this unit. Throughout the workday, RRP soldiers worked in the section, performing their first joint operational mission, and were integrated with the unit soldiers during breaks, sports activities and cultural events, such as celebrating holidays and going on tours. They were expected to prove their professional skills, and to be productive. Moreover, they needed to adapt their social communication skills, behavior and interests to the expectations of the army, as well as peers and colleagues unfamiliar with ASD. It is important to remember that the army is an inflexible system with very strict demands and setting. One might assume that at this stage, the participants may become overwhelmed and stressed, and thus, feel less satisfied with their quality of life. However, the results indicate a significant enhancement in the participants’ perception of subjective QOL following six months in the army unit, suggesting that the feeling of achievement, and specifically the participation within typical social environment and being a meaningful part of the community are factors which may have major importance and contribute to their sense of quality of life. The army phase may establish a stronger sense of independence and control, domains that are reflected at the QOL, which relates to real life occupation and status of independence and control. Indeed, the occupational therapy practice framework [2] identified QOL as one of the major outcome indicators of engagement in occupation. 

It is important to mention that the domain of community integration did not change significantly after the course or the placement at work. This may be due to the fact that although they were integrated into an inclusive army unit, participants worked in a partly segregated unit. It would be interesting to investigate participants’ sense of community integration post-integration into the different unit sections, about one year after enlistment. Another possible explanation of this finding may be the nature of service in the army as opposed to the questionnaire’s indicators for this domain. For example, the QOL-Q asks about activities in clubs in the community, dating and interactions with neighbors at home. Life in the army does not always enable such activities. It is possible, however, that these aspects of life, which are considered to represent good quality of life for typically-developed individuals, do not appeal to young adults with ASD who often, due to their unique interests and the poor socialization skills, may not chose to participate in typical free time activities, and therefore these items may not even represent quality of life for them. Indeed, theoreticians in the QOL area have called for the development of specific subjective QOL indexes for people with ASD due to their unique behavioral, communication and social characteristics to better assess their needs and perception on life [22]. 

### 4.2. Subjective Well-Being (SWB) Along the Vocational Program

The PWI is a frequently used global index of SWB that has been developed to measure subjective satisfaction from various life domains. It is directed to the general population, as opposed to measuring a specific and selected population. It allows the individual to project and relate to each domain from his or her point of view, as opposed to relating to specific indicators in each domain (International Wellbeing Group, 2006). It is important to note that prior to the training phase, the reported mean of the general question concerning SWB, “how happy do you feel about your life as a whole?” was around the reported means in the literature (73+/−3) [18]. As for the domains, they are within the normal distribution (50–100), however, level below 70 is considered to be at risk for imbalance in perception of SWB. The domain of safety in life was rated at the lower range. Safety is considered to indicate emotional well-being [16] and, in this study, it was the main domain that improved immediately after the training and the acquisition of the profession. The other life domains, as assessed by the PWI, showed fluctuations in the three points of testing, but did not reach statistical significance. 

These results can be due to the nature of measuring subjective well-being and the index we chose. As mentioned previously, the PWI is a global measurement of wellbeing. It asks the person about his or her perception concerning a general life domain, but does not go into details to assess the various indicators for each domain. Cummins hypothesized that subjective well-being represents more of an affective state than a cognitive state [23]. He also hypothesized that people are usually satisfied with their lives while their physical and social needs are met, and as a result of other studies, he developed his theory of Subjective Well-being Homeostasis. It hypothesizes that every individual has his or her own set point of personal well-being, like a body temperature. This point changes within a relatively narrow range and can be affected by life events, stress, *etc.* [24] and that interventions are crucially dependent on this set-point baseline. For people operating normally within their set-point-range (70+), an effective intervention will achieve a small increase in subjective well-being. However, for people who operate in the lower ranges or around 50 and below, successful intervention can be crucial. 

The current findings suggest that the participants operated in slightly lower than the 70% range (on a scale of 10 that is converted to 100%) and ultimately remained around it, except for the safety domain that increased significantly from its lower set point. Thus, in most of the PWI questionnaire domains, the reports of these young people with ASD were similar to those of the general population [18,25]. However, these results should be analyzed with caution, due to (a) the lack of norms for the Israeli general population, and (b) there is very little research on ASD and satisfaction from life in general, and specifically with this measure (PWI). 

In addition, these findings suggest a trend of improvement in the domains of future security and connectedness to the community, immediately after the course, and in personal relationship and self-determination after the army phase. Due to the small sample size, it is difficult to draw conclusions, and therefore further assessment is recommended. However, this trend is promising and is in line with the theory that stresses the importance of providing individuals with (intellectual developmental Disabilities (IDD) the opportunity to participate in a meaningful productive (work) and leisure activities [26].

Indeed, previous evidence indicates that job satisfaction is strongly and consistently related to subjective well-being, which may be due to a “spillover” of the experience at work onto life experience and *vice versa* [27]. 

## 5. Conclusions and Limitations

To summarize, the results of the current study indicate that rather than acquiring an applicable, appropriate and desired profession only, it is the actual application of the acquired skills and the transformation into meaningful job within the community that enhanced the perception of QOL and subjective well-being. These results support previous literature on young adults with ASD, which highlights the importance of work as it presents a primary aspiration of adolescents and young adults, as well as an important aspect of their independence, participation and successfully integration into the adult world [1]. 

Although the small sample size and the absence of a control group limit the generalizability of the study findings, they highlight the importance of vocational rehabilitation in the form of programs that are adapted to the unique needs and strengths of individuals with ASD. Such programs provide a major starting point for further programs and more research to follow. These courses open the doors to people with ASD to enjoy meaningful work, which affects their quality of life and subjective well-being.
